# The Impacts of the COVID-19 Pandemic on Service-Learning Experiences Among Undergraduate Public Health Students in Hawai‘i

**DOI:** 10.3389/fpubh.2022.771844

**Published:** 2022-04-28

**Authors:** Lisa Kehl, Uday Patil, Michelle Tagorda, Denise C. Nelson-Hurwitz

**Affiliations:** Office of Public Health Studies, Thompson School of Social Work and Public Health, University of Hawai‘i at Mānoa, Honolulu, HI, United States

**Keywords:** service-learning, undergraduate studies, public health, COVID-19, bachelor's degree, high-impact educational practices, BAPH, BSPH

## Abstract

Service-learning is a high-impact educational practice at the core of the undergraduate public health degree at the University of Hawai‘i at Mānoa (UHM). This practice provides an invaluable learning experience and professional opportunity for students to collaborate with community partners and make significant contributions in the field. The COVID-19 pandemic halted or disrupted service-learning experiences as community partners adapted to shifting mandates and emergency orders. Surveying the rapidly evolving landscape of partner organizations to support service-learning is a challenge. Assessing changes to the program mentorship or satisfaction is the first step to developing protocols to ensure standardization of service-learning during times of crisis. This study will address if and how the pandemic impacted students' satisfaction with required service-learning experiences. Furthermore, authors hope to create a comprehensive list of practicum partnering organizations, both focused on pandemic response and, more generally, of the service-learning students at UHM, with the intent to increase students and community partners in local service-learning. Assessments were conducted to assess the impact of COVID-19 on undergraduate students' experiences with service-learning through use of a program exit survey. The authors hypothesized pandemic-related adjustments would not affect student satisfaction or skill development. Despite challenges associated with the pandemic and emergency online transitions, students persisted in personal and professional growth associated with service-learning. This developed resilience supports students as they graduate and enter a workforce adapting to remote work demands and community needs.

## Introduction

The establishment of undergraduate programs in public health has been a recent development nationwide (1). Service-learning is a key component of these programs. Service-learning is a type of experiential learning that involves students providing a service to a community organization and the organization providing a learning experience for the student. The Health Professions Schools in Service to the Nation (HPSISN) program defines service-learning as “a structured learning experience that combines community service with explicit objectives, preparation, and reflection” (2). It is an academic activity in which students are applying skills learned in the classroom in the community (2). As a key tenet of undergraduate education, service-learning is 1 of 11 common high-impact educational practices (3).

The COVID-19 pandemic upended the education experience across the globe (4–8). Many states, including Hawai‘i, issued emergency stay-at-home orders, closing down non-essential businesses and schools, including universities (9, 10). On March 12, 2020, the University of Hawai‘i notified all 10 campuses that classes would move online for 2 weeks following spring break (11, 12). By the time classes resumed following spring break, in-person classes had been moved online for the remainder of the semester and the entire state was under a stay-at-home order. Educational programs were required to pivot their service-learning programs in response to the pandemic.

### Pandemic Impacts on Service Learning

The COVID-19 pandemic and associated mandates and regulations disrupted student service-learning experiences (6, 8, 13). Community organizations are, and continue to be, challenged by evolving requirements and emergency orders, leading to variable responses to student engagement requests. Meanwhile, students have limited service-learning options, and educational program responses need to be increasingly adaptive. Effective experiential learning programs are often faced with rapidly evolving situations and circumstances that impact students and partner organizations.

Many pandemic-related restrictions were put into place by government officials in the State of Hawai‘i (10, 14). Following strict public health guidance, limitations to in-person activities unpredictably affected community organizations that provide direct service to communities, especially where demand for such services is highly variable. Moreover, many organizations were projected to lose funding or other resources, reducing capacities to work with, and regularly mentor students.

The pandemic had immediate impacts on every organization—some were able to adapt quickly and continue providing services remotely, some were not able to pivot to remote services and instead reduced what they could offer clients, while some found their clients had new needs and designed new approaches to fill those needs (e.g., distributing food), and others still had financial issues and were forced to lay off the majority of their staffs. All of these scenarios had different effects on students and their service-learning activities.

### Applied Learning Experience

The Applied Learning Experience (APLE) is a capstone service-learning program requirement within the Bachelor of Arts in Public Health (BAPH) degree at the University of Hawai‘i at Mānoa (15). It is implemented through a three-course capstone series. During the APLE students conduct 100–120 h of service-learning with a community organization, typically completed over a semester or summer.

The conceptual frameworks on which the APLE is built atop consist of three major components: the Council on Education for Public Health (CEPH) competencies for undergraduate degree programs (16), the 10 Essential Public Health Services (EPHS) (17), and the landmark service-learning model developed by Yoder (18, 19).

By accrediting schools and programs of public health, not only do CEPH domains and competencies guide bachelors in public health learner outcomes, but also require a culminating experience to prove students are able to translate their new knowledge into effective work with community partners. As such, the required embedded service-learning opportunity offered to students also serves the ultimate goals of the program. But as Mackenzie et al. (20) report in a 2019 summary of a bachelor's-level service-learning experience, CEPH falls short in dictating how programs should meet those goals, and the group acknowledges that student learning builds upon itself in esoteric ways.

The EPHS, most recently revised in 2020 by Public Health National Center for Innovations (PHNCI) and the de Beaumont Foundation, summarizes the work of public health practitioners, researchers, and instructors into 10 fields, of which form three main categories: assurance, policy development, and assessment (17). This omnipresent framework directs practicum placements to clearly identify their potential services as one of the established public health services. Having unambiguous positions allows for the student and administrator to understand their value to the field (and report such to CEPH).

Under the Yoder model, four aspects to consider when developing service-learning opportunities include growth, scholarship, programs, and partnerships (18). The developed APLE articulates clearly with this model. For example, within scholarship, an academic link in APLE is that the service-learning is directly tied to an academic course, PH 485: Public Health Applied Learning Experience, and academic preparation for field experience in PH 480: Applications of Public Health in Research and Practice. While students are engaged in service-learning experiences, they are also supported and supervised by the Field Education Coordinator, a faculty position. The APLE program additionally has sustained community partnerships (a Yoder model component within the partnerships aspect) (19), with multiple non-profit organizations, government agencies, and university faculty that have been cultivated and sustained over time. Community partners will frequently collaborate with multiple students over time, in sequential semesters, demonstrating sustained partnership between the University and individual organizations.

#### Process

The APLE process starts first with a preparatory course, PH 480: Application of Public Health Principles in Research & Practice. In this course, usually taken the semester prior to PH 485, students conduct a literature review on an individually selected public health topic of interest and organizations involved in this topic. In the subsequent course, PH 485, a service-learning experience of 100–120 h is required. Students typically complete these hours over a 16-week semester or the summer break, consisting of 12 weeks. Students work with organizations individually to develop a mutually agreeable work schedule.

Throughout the process, and concurrent with the mentored field experience, students engage in writing reflective blogs and in participating in class discussions and check-ins. The third course in the APLE capstone series is PH 489: Public Health Undergraduate Capstone Seminar, where students develop an individual capstone report, which combines their academic preparation (i.e., topic literature review) with field experience gained through service-learning. Students generally take PH 489 in their final semester before graduation.

#### Pandemic Adjustments

In the spring of 2020, the COVID-19 pandemic began and classes at the University of Hawai‘i at Mānoa (UHM) were moved to an online format beginning on March 23, 2020. Service-learning immediately disappeared from most UHM classes that had previously included it as a course component, as in-person interactions were often not allowed under newly developed COVID-19 policies. In some cases, significant modifications were made to service-learning assignments, or they were eliminated altogether. Restrictions at UHM specified students could not be required to attend in-person activities as part of their course work.

At this time, all APLE students and their sites were notified that all activities and service-learning hours would be required to be completed remotely for the remainder of the semester. This brought a multitude of immediate challenges, as students and sites responded in different ways. Some were able to pivot and switch to remote service or collaboration, while others were unable to adapt and ended their mentorship.

As a school of public health in the middle of a public health crisis, many BAPH students felt called to help and rose to the challenges presented by the pandemic. They chose to take advantage of the tremendous opportunities created by the pandemic to apply public health skills in the community. There was no better time for service-learning and many students signed up to help volunteer with the Hawai‘i Medical Reserve Corps (MRC) (21) to give back to the community and help those in need.

At the Office of Public Health Studies (OPHS), direction for program development was taken from CEPH (22) regarding how to address students currently conducting service-learning projects as part of the requirements of their academic degree. CEPH provided guidance on their website and encouraged flexibility and creativity in developing solutions and projects for students to engage in: “Maximum flexibility and creative solutions should be applied to students whose experiences are impacted by the current crisis” (22). “CEPH supports flexible approaches at this time and you may temporarily modify your policies to accommodate current circumstances” (22). Using this guidance, minimal adjustments needed to made to the service-learning supervising course aside from communicating evolving University policy changes to the students and maintaining awareness of existing and emerging personal challenges among students.

### Study Purpose

During the COVID-19 pandemic, surveying the rapidly evolving landscape of service-learning opportunities and partner organizations has been a challenge. Assessing changes among community organizations of program mentorship and satisfaction is the first step to developing protocols to ensure standardization of service-learning during times of crisis. More generally, there is little known about the challenges faced by this high-impact educational practice during COVID-19, and even less reported on unexpected successes during this transition.

This study will address if and how the pandemic impacted students' satisfaction, skill development, and perceived support to future goals with the Applied Learning Experience. Furthermore, the authors hope to create a comprehensive list of partnering community organizations, both focused on pandemic response and, more generally, of the service-learning students at UHM, with the intent to highlight community partner responses to local service learning during a global pandemic. The authors hope to glean valuable insight into student satisfaction, skill development, and perceived support associated with service learning during this pandemic and implications for service-learning opportunities during future crises, locally and globally.

## Methods

### Study Design and Sample

The proposed study examines a cohort of BAPH students focusing on the APLE portion of their academic journey. It will compare students who conducted their APLE during calendar year 2019 to students who conducted their APLE during calendar year 2020. The students in the 2019 cohort will serve as a baseline (Pre-COVID), while the students in the 2020 cohort will serve in one of three comparison groups, depending on which pandemic-related stage the students participated in service learning. These stages include (a) Spring 2020-COVID Interrupted; (b) Summer 2020-COVID Impacted; and (c) Fall 2020-COVID Adapted. While the pandemic continues to evolve and subsequent semesters' of service-learning may also be considered as COVID Adapted, the scope of this study is limited to COVID-19 impacts in 2020.

### Measures

The primary data source is the OPHS BAPH Exit Survey (see Appendix A), a Google Forms survey that collected data on student satisfaction with the APLE, and ratings of service-learning quality with regard to development and application of public health skills, among other items. This data source was linked to students' names and/or email addresses so that data from different sources can be combined to create a profile for each student.

Service-learning partner organizations that participated during the study timeframe have been listed in Table 1. Partner organizations have been identified as locally based and/or nationally based with local affiliation. Also, those agencies with specific COVID-19 pandemic response programs have been noted.

**Table 1:** Service-learning partner organizations.

**Name of organization**	**Local organizations, specific to Hawai‘i**	**National organizations with local affiliates**	**COVID-19 response organizations**
American Cancer Society-Cancer Action Network Hawai‘i-Pacific		X	
American Lung Association in Hawai‘i		X	
Blue Zones Project Hawai‘i	X		
Department of Health, Medical Reserve Corps Oahu	X		X
Hawai‘i Health & Harm Reduction Center	X		X
Hawai‘i Public Health Institute	X		X
Healthy Mothers, Healthy Babies	X		X
John A. Burns School of Medicine, Department of Psychiatry	X		
Kalihi Kidz	X		
Kokua Kalihi Valley	X		X
Mental Health America of Hawai‘i		X	
Our Kupuna	X		X
Palama Settlement	X		X
Surfrider Foundation Hawai‘i		X	
University Health Services Mānoa, Health Promotion	X		
Waianae Coast Comprehensive Health Center	X		X
YMCA of Honolulu		X	

This study was reviewed and approved by the University of Hawai‘i at Mānoa. It was deemed to not be human subjects research.

### OPHS BAPH Exit Survey

This Google Forms survey was distributed via email from OPHSAS to all students at the end of the semester that they are enrolled in PH 489, typically the semester they graduate. This data has been collected every fall and spring semester since the BAPH program graduated its first students in 2015.

### Analysis Plan

Data from the OPHS BAPH Exit Survey were transferred from the Google Forms survey platform to Microsoft Excel. All sources of data were coded into Pre-COVID, COVID Interrupted, COVID Impacted, and COVID Adapted, based on identifying information such as student names and email addresses, and correlated with PH 485 course enrollment data. Analyses of variance were used to evaluate differences among cohorts of students.

## Results

Table 1 lists service-learning partners, their geographical scope, and any response to the COVID-19 pandemic. In addition to these partners preexisting community organizations were also available for students to complete virtual service-learning experiences. Roughly one to five students were placed with available organizations during any given semester throughout the duration of the study. The multitude of service-learning opportunities attests to the wide community engagement of OPHS and willingness to train BAPH students to assist beneficiaries in the present moment, and to develop into effective staff members later. Service-learning experiences allowed students to practice all 10 essential public health services, helping alleviate issues ranging from poor nutrition to sedentary living to maternal mortality and serious mental illness.

While relatively small, the State of Hawai‘i offers many services to the sick, indigent, uninsured, and vulnerable. Hopefully, sharing this list will encourage other academic and professional providers of service-learning opportunities to forge partnerships with these social service agencies or contribute to the list.

### Participation and Response Rate

Of 100 students who were invited to participate in the Exit Survey, data were gathered, coded, and analyzed from 95 graduating BAPH students, all of whom graduated between Fall 2019 and Fall 2021. Thirty-eight students graduated in December 2019 and this cohort was used as a Pre-COVID comparison group. Twenty-three of these students conducted their APLE in Spring 2019 and 15 conducted APLE in Summer 2019. Data from each student's service-learning experience was available for analysis. There were 62 students who participated in service-learning experiences either interrupted (25 students), impacted (18 students), or adapted (19 students) to COVID-19, of which 57 responded to the BAPH Exit Survey (response rate: 91.94%; 3 students impacted by COVID and 2 students in COVID adapted had not graduated or had not responded to the BAPH Exit Survey). These data are summarized in Table 2.

**Table 2:** Sample size and response rates of exit survey data, reported by PH 485 student enrollment semester.

**Impact of COVID-19 on service-learning experience**	**Number of students**	**Student exit survey data (response rate)**
Pre-COVID (baseline/comparison)	38	38 (100%)
COVID interrupted	25	25 (100%)
COVID impacted	18	15 (83.3%)
COVID adapted	19	17 (89.5%)
All	100 students	95 (95.0%)

In the COVID Interrupted group (Spring 2020), all 25 graduating students provided adequate data through the Exit Survey. In the COVID Impacted group (Summer 2020), 15 of 18 (83.3%) students provided adequate data, and in the COVID Adapted group (Fall 2020), 17 of 19 (89.5%) students provided adequate data. In total, responses from 95 students (95.0%) were included in analyses of student satisfaction and service-learning opportunity quality.

### Student Satisfaction Outcomes

Table 3 summarizes student satisfaction and opportunity quality of practicum partner organizations. Over the pandemic stages (i.e., Pre-COVID, Interrupted, Impacted, Adapted), analyses fail to find statistically significant differences in perceptions that the APLE prepared students for the future: *F*_(3, 96)_ = 0.532, *p* = 0.661; adequate assistance was received in the development and supervision of the APLE: *F*_(3, 96)_ = 1.026, *p* = 0.385; the APLE helped develop skills in basic public health concepts: *F*_(3, 96)_ = 1.589, *p* = 0.197; and the APLE allowed for demonstration of the application of public health skills: *F*_(3, 96)_ = 0.324, *p* = 0.808. That is to say, students' perceptions were not significantly different as APLE experiences changed throughout the pandemic. While minor dips in satisfaction and opportunity quality are present in students engaging in service-learning at the start of the pandemic, these figures rebound as social service agencies adapted to operating during the health crisis. Moreover, these variations are not practically meaningful in a sample of this size.

**Table 3:** Summary of exit survey data, reported by PH 485 student enrollment semester.

**Impact of COVID-19 on service learning experience**	1a: How adequate was the Applied Learning Experience (APLE) that you gained here for preparation for your future?^*^	1b: How adequate was the assistance you received in the development and supervision of the APLE of your program?^*^	2: My APLE allowed me to develop skills in basic public health concepts^**^	3: My APLE allowed me to demonstrate the application of my public health skills^**^
Pre-COVID (baseline/comparison) (*n* = 38)	% Excellent: 65.8% Mean: 4.5 Median: 5.0	% Excellent: 57.9% Mean: 4.4 Median: 5.0	% Strongly Agree: 63.2% Mean: 4.6 Median: 5.0	% Strongly Agree: 55.3% Mean: 4.5 Median: 5.0
COVID interrupted (*n* = 25)	% Excellent: 60.0% Mean: 4.5 Median: 5.0	% Excellent: 72.0% Mean: 4.7 Median: 5.0	% Strongly Agree: 52.0% Mean: 4.5 Median: 5.0	% Strongly Agree: 52.0% Mean: 4.5 Median: 5.0
COVID impacted (*n* = 15)	% Excellent: 44.4% Mean: 4.3 Median: 4.0	% Excellent: 61.1% Mean: 4.6 Median: 5.0	% Strongly Agree: 50.0% Mean: 4.5 Median: 4.5	% Strongly Agree: 61.1% Mean: 4.6 Median: 5.0
COVID adapted (*n* = 17)	% Excellent: 52.6% Mean: 4.4 Median: 5.0	% Excellent: 73.7% Mean: 4.7 Median: 5.0	% Strongly Agree: 47.4% Mean: 4.2 Median: 4.0	% Strongly Agree: 47.4% Mean: 4.4 Median: 4.0
All (*n* = 95)	% Excellent: 58.0% Mean: 4.4 Median: 5.0	% Excellent: 65.0% Mean: 4.6 Median: 5.0	% Strongly Agree: 55.0% Mean: 4.5 Median: 5.0	% Strongly Agree: 54.0% Mean: 4.5 Median: 5.0

* *1 = Poor, 3 = Fair, 5 = Excellent*.

** *1 = Strongly Disagree, 3 = Neutral, 5 = Strongly Agree*.

Most students were highly satisfied with their service-learning opportunities. They rated the APLE with a median score of Excellent, the highest rating, with regard to how well it prepared them for future opportunities. Moreover, a median rating of Excellent was given with regard to the assistance received in the development and supervision of the APLE. The median scores did not differ between groups; that is, no significant differences were found in the satisfaction of APLE experiences before, interrupted by, impacted by, or adapted to the pandemic. Students also acknowledged that their practica allowed them to develop skills in basic public health concepts, with a median rating of Strongly Agree. More than half (55%) strongly agreed with the statement, and 54% strongly agreed that the APLE allowed them to apply public health skills with a median rating of Strongly Agree. These scores did not differ significantly among groups either.

On average, 58% of students responded that the service-learning experience prepared them for their future to an excellent degree, while 65% noted having an excellent level of assistance in the development and supervision of their APLE. In terms of opportunity quality, 55% of students stated they strongly agree that the APLE allowed them to develop skills in basic public health concepts and 54% strongly agreed with the assertion that the service-learning opportunity allowed them to demonstrate the application of public health skills.

## Discussion

Service-learning is an invaluable experience for students, which is not diminished by the many challenges imposed by a global pandemic. This high-impact educational practice has shown itself to be both flexible and resilient to continual policy changes and government interventions, including business shut-downs, curfews, and stay-at-home orders.

During the COVID-19 pandemic, students reported meaningful and educational service-learning experiences, both in-person and through remote learning modalities. Students were able to apply skills and knowledge gained in the classroom into practice in the community, while also developing new skills and rising to support emerging community needs. They did not miss important lessons or engagement opportunities because of community restrictions, but were flexible and adapted to changes and evolving challenges. Through pandemic-impacted service-learning, students found new ways to interact and learn through remote technology and participate in public health crisis response. Students who participated in these experiences are now better equipped to adapt to future challenges because of this experience and are more prepared to succeed in the workplace in the new normal of remote working.

Community organizations also demonstrated resilience and adapted to community needs, which further enabled students to make substantial contributions to the pandemic relief efforts by assisting with food distribution and early contact tracing, helping at COVID-19 testing sites, and engaging in online outreach efforts. Students directly not only witnessed harsh community disparities first-hand, but also saw the strength, dedication, and creativity of community organizations to modify their services and pivot toward addressing existing and newly exposed disparities, making this a uniquely valuable service-learning opportunity.

### Strengths and Limitations

This research study is the first to examine the impact of the COVID-19 pandemic on the state of service-learning within a university that provides practicum statewide. The novel analytic procedure identified three unique stages—COVID-19 Interrupted, Impacted, and Adapted—in which applied learning experiences were changed and community organizations were compelled to pivot. This framework can be tailored and utilized in future studies seeking information on student outcomes during the ongoing pandemic.

One of the limitations of this study was the use of secondary data, which was self-reported, rather than objectively measured. Moreover, the small overall sample size and group sizes limit the impact and generalizability of any results. Response bias should also be noted as a limitation. Those graduates with better experiences in the BAPH program (and APLE) may be more likely to respond to the exit survey. It is also possible that given the one semester break and activities associated with PH 489 students may respond with a more or less favorable representation of their service-learning experience which occurred in the previous semester. Socially desirability may also be present as students may be reluctant to provide negative reviews of practicum supervisors or BAPH advisors and instructors.

## Data Availability Statement

The raw data supporting the conclusions of this article will be made available by the authors, without undue reservation.

## Ethics Statement

The studies involving human participants were reviewed and approved by University of Hawai‘i at Mānoa. The Ethics Committee waived the requirement of written informed consent for participation.

## Author Contributions

LK conceptualized and designed the project. DN-H, MT, and LK implemented tools and collected data. LK and UP wrote the first draft of the manuscript. All authors wrote sections of the manuscript, contributed to manuscript revision, read, and approved the submitted version.

## Conflict of Interest

The authors declare that the research was conducted in the absence of any commercial or financial relationships that could be construed as a potential conflict of interest.

## Publisher's Note

All claims expressed in this article are solely those of the authors and do not necessarily represent those of their affiliated organizations, or those of the publisher, the editors and the reviewers. Any product that may be evaluated in this article, or claim that may be made by its manufacturer, is not guaranteed or endorsed by the publisher.

## Appendices

### Appendix A. OPHS BAPH Exit Survey (Excerpt)

1. Please rate the following on a 5-point scale (1 = Poor, 3 = Fair, 5 = Excellent)
a. How adequate was the Applied Learning Experience (APLE) that you gained here for preparation for your future?
b. How adequate was the assistance you received in the development and supervision of the APLE of your program?
2. My APLE allowed me to develop skills in basic public health concepts (1 = Strongly Disagree, 3 = Neutral, 5 = Strongly Agree).
3. My APLE allowed me to demonstrate the application of my public health skills (1 = Strongly Disagree, 3 = Neutral, 5 = Strongly Agree).
